# Reproductive Strategies in Paternal Care and Remarkably Low Paternity Level in a Giant Water Bug

**DOI:** 10.1002/ece3.71316

**Published:** 2025-04-23

**Authors:** Tomoya Suzuki, Shin‐ya Ohba, Koji Tojo

**Affiliations:** ^1^ Faculty of Science Shinshu University Matsumoto Nagano Japan; ^2^ Faculty of Human Environmental Studies Hiroshima Shudo University Hiroshima Japan; ^3^ Biological Laboratory, Faculty of Education Nagasaki University Bunkyo Nagasaki Japan; ^4^ Institute of Mountain Science Shinshu University Matsumoto Nagano Japan

**Keywords:** Belostomatidae, cuckoldry, maternity, mating trial, SSR marker

## Abstract

Reproductive strategies are crucial for organisms because they directly affect the organisms' fitness. “Parental care” is one of the strategies adopted by organisms to improve their fitness. However, even in the case of parental care, females often bear a large burden in raising offspring, and it is extremely rare for males to care for offspring alone (“paternal care”), especially among invertebrates. Offspring care results in fewer re‐mating opportunities, resulting in a greater reproductive cost for males. Under such conditions, paternal care has evolved in males of several animal taxa. However, there is a lack of clarity on how paternal care behavior has evolved and how it is maintained. In this study, we allowed males and females of *Appasus japonicus* , a typical paternal care‐exhibiting insect, commonly known as a giant water bug, to mate freely in the laboratory. We also used sensitive molecular markers that we developed for the giant water bug to conduct paternity testing trials. The results of these trials showed unexpectedly low actual paternity rates. Moreover, most males cared for other male's eggs, often from multiple males, and “cuckoldry” was evident. These discoveries constitute a significant finding regarding the evolution of reproductive strategies in insects because the paternity rate of belostomatid insects had been considered to be high. We provide new insights that overturn the established theory of the evolution of paternal care in insects.

## Graphical abstract

In this study, we conducted a mating trial in a laboratory for a typical paternal care‐exhibiting insect, *Appasus japonicus* , commonly known as the giant water bug. We also used sensitive molecular markers that we developed for the giant water bug to conduct a “paternity testing” experiment. The results of the experiment showed unexpectedly low actual paternity rates, and “brood parasitism” between males was evident.

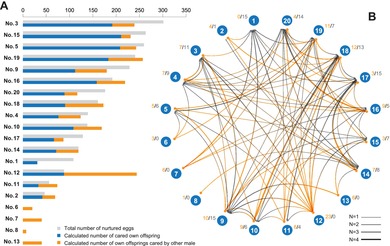

## 1 Introduction

DNA is the code of life, and passing on one's DNA to offspring is the most important and fundamental role of any organism (Dawkins 1976). With time, different organisms have developed diverse strategies to replicate their genetic information effectively so that sharing of genetic information is optimized, enabling the maintenance of genetic lineages. Organisms evolved different reproductive strategies through natural selection (Hirshfield and Tinkle 1975; Skinner 1981). Among the various reproductive strategies, parental care, which refers to caring for offspring directly, is the most common behavior exhibited by organisms to increase their fitness (Kölliker et al. 2012). Some forms of parental care behavior are exhibited predominantly by females (i.e., maternal care), while other forms in which caring behavior is performed by females and males together (i.e., biparental care) (Ridley 1978). While caring forms vary widely and depend on taxonomy, in general, female care is more widespread than male care (Kokko and Jennions 2008). The reason why male caring behavior is comparatively not as common as that of the female is that for males, increasing mating frequency is adaptively more advantageous than caring for offspring because there is a significant difference in the cost of gamete formation between males and females, and usually, the cost is comparatively lower in males (Kokko and Jennions 2003). Despite this trend, single‐handed caring behavior of offspring (“paternal care”) has evolved in the males of several animal taxa (Ridley 1978).

Paternal care behavior is relatively common among fish and is also reported among amphibians and birds (Ridley 1978). It is believed that a high degree of confidence in a male's paternity is required for the evolution of paternal care (Smith 1979). For example, after female seahorses lay eggs in the males' “brood pouch,” the males fertilize the eggs inside their pouch and “deliver” their juveniles (Jones and Avise 2001). Therefore, their certainty of paternity is absolute. Several studies that elucidated the evolution of paternal care systems focused on how males maintain high certainty of paternity (Ridley 1978; Smith 1979; Kokko and Jennions 2003; Lehtinen and Nussbaum 2003; Ah‐King et al. 2005). However, recent studies have revealed that high male certainty of paternity is not an absolute requirement for the evolution of every paternal care system (Kamel and Grosberg 2012; Rueger et al. 2019; Valencia‐Aguilar et al. 2020). For example, Brazilian harvestman females choose males who display paternal care behavior as mating partners (Nazareth and Machado 2010). Furthermore, in this species, males are willing to adopt genetically unrelated eggs, probably because the presence of eggs increases males' mating success. This finding reinforces the argument that certainty of paternity is not required for the evolution of paternal care. It also supports the hypothesis that the egg itself is the criterion by which females choose mates (Alonzo 2012), aligning with the view that high certainty of paternity is not necessary for the evolution of paternal care behavior (Tallamy 2001).

While insects are the most diverse group of organisms on earth from the viewpoint of species diversity, only 0.015% of insect species have evolved paternal care strategies (Smith 1997). Almost all the insect species showing paternal care behavior belong to the giant water bug family, Belostomatidae (Smith 1997). Specifically, a unique paternal care system has evolved in species of the subfamily Belostomatinae, where females lay eggs on the males' backs and the males care for the egg mass until it hatches (Smith 1997). In addition, males do not allow oviposition without copulation, and mating is performed prior to the oviposition of each egg until the egg mass formation is completed (Smith 1997). This behavior has been considered as the male's strategy for improving certainty of paternity (Smith 1997).

While giant water bugs have a highly unique reproductive strategy, the relationship between certainty of paternity and their mating system is unresolved. Female giant water bugs have spermatheca, and their reproductive mode is promiscuous; thus, it is suggested that there is sperm competition (Inada et al. 2011). Moreover, paternal care behavior of this group is necessary for hatching, and males care for multiple egg masses during each reproductive season (Ohba 2019). There are high subordinate costs of paternal care (e.g., loss of flight ability and increased risk of predation due to the protrusion of the egg mass on their back), in addition to the caring behavior itself (Crowl and Alexander 1989; Kight et al. 1995). Under these conditions (i.e., a promiscuous‐type mating system, potential sperm competition, and high costs of paternal care), it is possible that males mate and deposit their sperm in females without them performing paternal care.

Furthermore, it has been suggested that (1) males carrying more eggs on their dorsum, regardless of whether the eggs were fertilized by themselves or by other males, increase the number of their own eggs being fertilized by enhancing mating opportunities (see Nazareth and Machado 2010; Ohba et al. 2016), and (2) when paternal care imposes high energetic costs on males, increased investment in parental effort may limit the resources available for fertilization effort, thereby leading to a higher likelihood of paternity loss due to cuckoldry (Requena and Alonzo 2017). Based on these previous hypotheses, it is likely that male belostomatine insects that care for a large number of eggs gain more mating opportunities and produce a greater number of offspring. However, at the same time, the state of cuckoldry may result in reduced male paternity. In this study, we conducted a free‐choice mating trial using 20 pairs of *Appasus japonicus* over one month under laboratory conditions to confirm their paternity using microsatellite markers. Based on these results, we examined the following: (1) whether males carrying a large number of eggs on their backs, regardless of whether the eggs were fertilized by their own sperm or another male's sperm, gain more mating opportunities and increase the number of eggs they fertilize; and (2) whether paternal care in *A. japonicus* imposes high costs on males and, given the promiscuous mating system, leads to intense sperm competition within the female's spermatheca, thereby increasing the likelihood of paternity loss and resulting in a state of cuckoldry with reduced male paternity.

## 2 Materials and Methods

### 2.1 Sampling

We collected 20 pairs of *A. japonicus* before the reproductive season in mid‐April 2012 in Matsumoto, Nagano, Japan (N36.292435, E137.990177). The organisms were maintained in the laboratory at room temperature. Males and females were maintained separately in containers [*ca*. 11.5 L (365 × 210 × 150 mm)] until they were ready to copulate. A sufficient number of mayfly nymphs (over 100 individuals, mainly species of Heptageniidae, Ephemeridae, and Siphlonuridae) was given as prey each day.

### 2.2 Free‐Choice Mating Trial in the Laboratory

We conducted a free‐choice mating trial in a laboratory, as it is difficult to obtain individual data in the field. Twenty pairs of *A. japonicus* were used for the free‐choice mating trial in the laboratory. We used a plastic container [*ca*. 11.5 L (365 × 210 × 150 mm)] to conduct the mating trial. Before the experiment, we estimated the population density of this species at the collection site using the “Catch‐Effort” method (Hayne 1949): we established a 2‐m sampling area at the study site and conducted three 5‐min quantitative samplings within this area. Collected samples were removed from the sampling area, and population density was estimated based on the rate of decline in the number of individuals each time. As a result, approximately 210 individuals were estimated to be present within the 2‐m sampling area. Based on this result, we conducted the mating trial under the same population density conditions as in the field. The population density in this collection site was also preliminarily estimated using the “Mark‐Recapture” method, and the results obtained were similar to those obtained using the “Catch‐Effort” method. The sex ratio of this population was 1:1. The mating trial period was between June 28, 2012 and August 10, 2012, which is the core reproductive season of this species.

**FIGURE 1:**
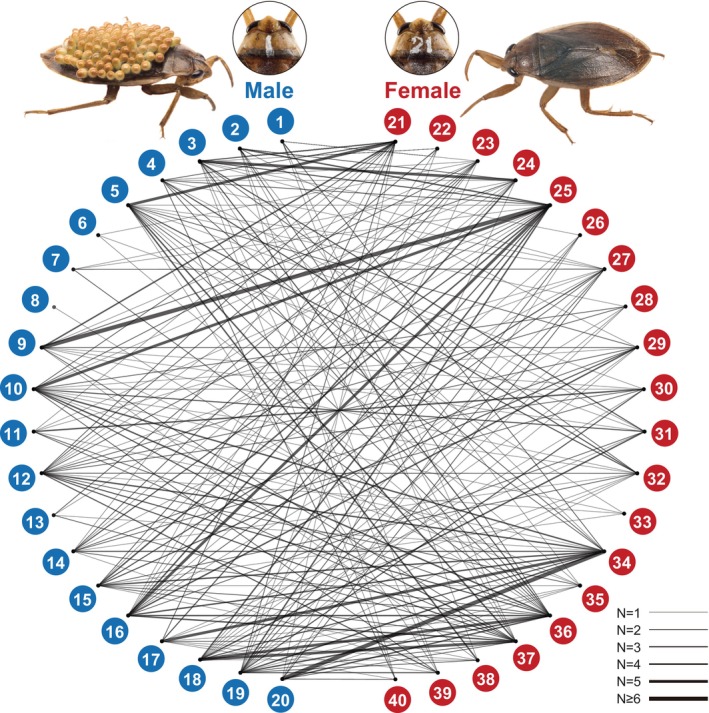
Relationships between copulated pairs based on their simple‐sequence repeat data. The connecting lines indicate the copulated pairs. The width of the line indicates the number of offspring of each pair. The blue and red circles indicate males and females, respectively, and the number in the circles indicates their individual identification (ID). The reproductive experiment was conducted using individuals with their IDs written on the dorsal anterior thorax, as shown in the photos of specimens No. 1 and No. 21.

We arranged branches as perches in a container, with the number of perches exceeding the number of individuals used in the experiment (*n* = 20). We used tap water kept overnight for our experiment, and the water depth in the container was set to approximately 100 mm. We wrote individual IDs using a permanent marker (Pentel WHITE 100 W S; Pentel Co. Ltd., Tokyo) on the dorsal part of the thorax of all individuals used in the mating trial in order to ensure individual identities (Figure 1). Typically, the egg mass is fully formed within a single day, after which the male does not allow the oviposition of additional eggs. We checked the individuals every day to see whether the males had commenced paternal care or not. Males that had commenced paternal care during the mating trial period were kept individually in separate plastic containers [*ca*. 0.36 L (111 × 78 × 42 mm)] within 24 h from when the male commenced paternal care. The number of eggs that a male can carry on its back is approximately 100 (maximum), and the embryogenesis period is approximately 2 weeks under 25°C. In this species, males drop the hatched empty egg mass in order to then carry the next egg mass on their back. The males typically conduct paternal care several times within one season. After the paternal care period was complete (eggs hatched), males were returned to the plastic container in which the free‐choice mating trial was being conducted. Identities were assigned to each egg mass and kept with information on the corresponding care‐providing male. The number of eggs in each egg mass was also recorded. The hatched nymphs of each egg mass were fixed and kept in separate vials of 100% EtOH for use in molecular genetic analyses (i.e., all hatched nymphs from one egg mass were kept in one vial).

### 2.3 Microsatellite Analysis and Genotyping

We conducted a microsatellite analysis using all adults and 10 nymphs from each egg mass. Thirty‐nine egg masses were cared for by the males during the experimental period [1.95 egg masses/male on average (range, 0–3)]. Therefore, 40 adults and 390 nymphs were analyzed. We randomly selected the analyzed samples to reduce any bias due to the egg's position within an egg mass. We used 20 microsatellite marker sets that we had previously developed (Suzuki et al. 2021). We used the same PCR protocol and polymerase as those previously described by Suzuki et al. (2021). A 3130xl capillary sequencer (Applied Biosystems, Waltham) was used for fragment analysis, and GeneMapper (Applied Biosystems, Waltham) was used for genotyping. The fragment analysis of all microsatellite markers for all samples was repeated three times in order to obtain robust data.

### 2.4 Verification of Paternity and Maternity

We searched for the existence of null alleles within each locus using MICRO‐CHECKER v2.2.3 (Van Oosterhout et al. 2004) and removed loci that included null alleles when verifying the paternity and maternity of each offspring. Therefore, we used 17 loci for verification. Verification was performed using Cervus v3.0 (Kalinowski et al. 2007) based on the following factors: the estimated number of alleles at the locus (*k*), number of individuals typed at the alleles (N), observed heterozygosity (*H*o), expected heterozygosity (*H*e), polymorphic information content (PIC), average non‐exclusion probability for one candidate parent (NE‐1P), average non‐exclusion probability for one candidate parent given the genotype being known of the other parent (NE‐2P), average non‐exclusion probability for a candidate parent pair (NE‐PP), average non‐exclusion probability for the identity of two unrelated individuals (NE‐I), and average non‐exclusion probability for the identity of two siblings (NE‐SI). In the present study, all males and females were entered as candidate fathers and mothers in Cervus v3.0 software (Kalinowski et al. 2007), and the possibility of the existence of other candidate parents when performing the verification was excluded because we conducted the free‐choice reproductive experiment under laboratory conditions. We performed the parental verification of each egg mass, and if a female died by the time a male commenced paternal care, we excluded these females as candidate mothers.

### 2.5 Rates of Parental Verification of Paternity and Cuckoldry

Thirty‐nine egg masses were cared for by the males during the experimental period, and parental verification was performed for 390 nymphs. Paternity and cuckoldry rates were calculated from the results of parental verification of 390 specimens.

The overall paternity rate for each egg mass was determined by calculating the proportion of the male's own offspring among 10 randomly sampled individuals from that egg mass. The cuckoldry rate for each male was calculated as the number of its offspring that hatched without its direct care divided by the total number of offspring examined (390 individuals in total).

After estimating the paternity rate and cuckoldry rate, we calculated the total number of offspring for each male during the free‐choice mating trial. (1) The total number of self‐cared‐for offspring for each male was determined by applying the paternity rate to the total number of cared‐for nymphs hatched from an egg mass. (2) The total number of offspring hatched through other males' caring for each male was calculated by applying the cuckoldry rate to the total number of hatched nymphs (2640 individuals in total) during the free‐choice mating trial.

### 2.6 Statistical Analysis

We hypothesized that male that care more eggs on their dorsum irrespective of own‐ or other male's‐fertilized eggs increase own fertilized‐eggs throughout increasing mating opportunity (see Nazareth and Machado 2010; Ohba et al. 2016). To test this hypothesis, we made two models incorporating the number of the male's fertilized offspring cared for on the male's dorsum (e.g., direct cared offsprings) or the number of offspring overall in the mating trial (= the total number of the male's own offspring fertilized on the male's‐ and other male's‐dorsum) as response variables. Regarding these response variables, the values showed overdispersion. Therefore, we used a generalized linear model [GLM; glm.nb in *MASS* package (Venables and Ripley 2002)] with a negative binomial distribution, incorporating the total care eggs (TCE; the total number of own‐ and other male's‐fertilized eggs cared for by each male), the male body size (prothorax width, mm), and their interaction as an explanatory variable. All models were compared and ranked according to Akaike's Information Criterion (AIC) in the *MuMIn* package in R (Barton and Barton 2013). This method compares the explanatory ability of each model using Akaike weights, which may be interpreted as the probability that a given model is the most likely description for the observed data (Burnham and Anderson 2002). Model comparisons were based on their delta AIC, which is the difference between the AIC for each model and the lowest observed AIC value (delta AIC = 0 indicated the ‘best’ model). Models with AIC values differing by less than 2 were considered to be equivalent (Burnham and Anderson 2002).

## 3 Results

### 3.1 Microsatellite Genotyping and Polymorphism

The results of genotyping are shown in Table S1. Almost all loci were successfully genotyped in all analyzed samples except the microsatellite marker AJP43 (Table S1). The missing alleles were treated as “0” (Table S1). The highest number of alleles was observed in the locus AJP10 (*k* = 6), and its observed heterozygosity (*H*o) and expected heterozygosity (*H*e) were 0.551 and 0.513, respectively (Table S2). The lowest number of alleles was observed in the loci AJP01, AJP04, and AJP43 (*k* = 2) (Table S2). The highest heterozygosity was observed in the locus AJP47, and its *H*o and *H*e values were 0.626 and 0.595, respectively (Table S2). The lowest heterozygosity was observed in the locus AJP28, and its *H*o and *H*e values were 0.002 and 0.007, respectively (Table S2).

### 3.2 Mating Trial, Verification of Paternity and Maternity

**FIGURE 2:**
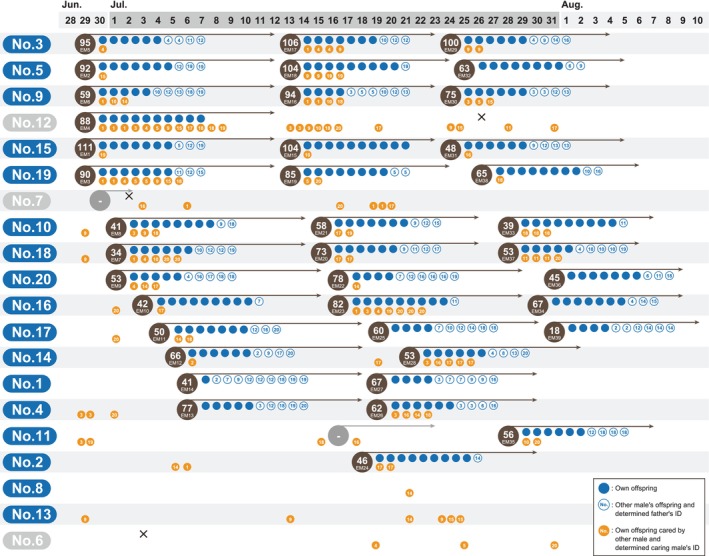
Results of the paternity test of each egg mass. Individual identifications (IDs, 1–20) are shown on the left side, and the dates of the experimental period are shown along the top. The male's IDs were sorted by the date of the start of the paternal care. The brown circles, which are shown on the row of data corresponding to each male, are positioned to indicate the date on which paternal care began, and the numbers shown in the upper and lower parts of the brown circles indicate the number of eggs cared for and the egg mass ID, respectively. The length of the arrow extended from the brown circle indicates a male's paternal care period. Gray circles indicate an egg mass that did not hatch due to death of the male carer or being dropped by the male before hatching. Cross marks indicate the death of a male. The circles filled with blue indicate each male's own offspring. The open circles with a number indicate offspring cared for that were not that male's offspring, and the numbers indicate the actual father's ID. The circles filled with orange indicate the eggs, which were hatched by other male's care, and the number inside indicates the carer male's ID.

**FIGURE 3:**
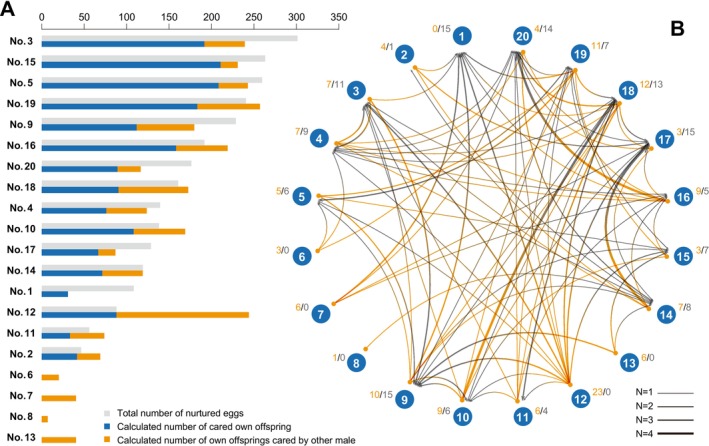
Total number of cared eggs, number of each male's offspring, and relationships between the males cared by other male's egg. (A) The gray bar of the left graph indicates the total number of eggs that each male cared for during the experimental period. The blue and orange bars indicate the number of own offspring and the number of cuckolded offspring that were hatched, respectively. These values were determined using data of the paternal and cuckolded egg counts calculated after analyzing individual insect's DNA. (B) Relationship between each male and his cuckolded offspring; the arrows indicate number of eggs cared by other male. The two numbers shown to the side of the individual IDs indicate the total number of offspring that were cuckolded for other males, that is, number of cuckolded offspring (orange), and number of offspring that were cuckolded by other males (gray).

**FIGURE 4:**
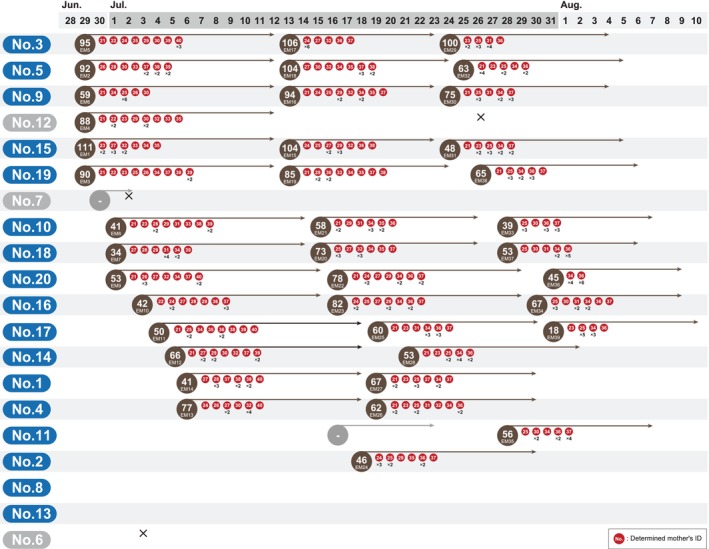
The mother determined to be responsible for each egg mass. This figure is interpreted as Figure 2 except for the red circle. Sampling was conducted using 10 offspring from each egg mass, and the number in each red circle indicates the female's ID. Numbers shown under the red circle indicate numbers of the 10 sampled offspring that were laid by the same female.

The results of the verification of paternity and maternity indicated that almost all males and females copulated, except male No. 8, and females left offspring with multiple mates (Figure 1). Six males (No. 3, 5, 9, 12, 15, and 19) began paternal care on the second day of the mating trial (Figure 2). Almost all males began paternal care by 9 days after the mating trial started. In addition, almost all males began caring for a second egg mass the next day after the first paternal care finished (Figure 2), and most males cared for at least one egg mass during the mating trial (Figure 2). Males No. 6, 8, and 13 did not conduct paternal care during the mating trial (Figure 2). The largest number of eggs cared for by males was 111 (Male No. 15, egg mass ID: EM1), and the fewest number of eggs cared for by males was 18 (Male No. 17, egg mass ID: EM39) (Figure 2). There was a variation in the males' paternity in our mating trial, and the calculated highest paternity was 100% in the egg mass ID EM4 and EM15, which was cared for by males No. 12 and 15, respectively (Figure 2). The calculated lowest paternity was 10% in the egg mass ID EM14, which was cared for by male No. 1 (Figure 2). The calculated mean paternity was 65.1%. Cuckoldry was detected in almost all males by our paternity verification (Figures 2 and 3). Males No. 6, 7, 8, 12, and 13 cuckolded others, as they mated without providing parental care, but they were themselves not cuckolded, as they did not care for the eggs of other males (Figures 2 and 3). Our calculation of the total number of eggs for cared and cuckoldry based on the paternity of each male indicated that male No. 3 cared for the largest number of eggs, and male No. 19 left the largest number of own offspring (Figure 3A). The maternity verification indicated that all egg masses were formed by multiple females (Figure 4). The largest number of females as mothers in an egg mass was detected in the egg mass ID EM3 (nine females were detected as mothers) (Figure 4). Detailed results of paternity and maternity verification (Identified mother, Identified father, Trio LOD score, and Trio Delta values) of each sample are shown in Table S3.

### 3.3 Does a Male Caring More Eggs Irrespective of His Own and Others' Fertilized Eggs Increase His Fitness?

For the two response variables (the number of the male's fertilized offspring cared on the male's dorsum or the number of offspring overall in the mating trial), two best models were each selected based on AIC (Tables S4 and S5). In the best models, the estimated coefficients of total care eggs (TCE; the total number of own‐ and other male's‐fertilized eggs cared for by each male) were positive. The 95% confidence intervals (CI) of all of the estimated coefficients of the ‘TCE’ did not include the value zero in both models (Tables S6 and S7), whereas those of ‘body size’ did. Table S6 indicates that the higher the number of eggs cared for, including eggs fertilized by other males' sperm, the higher number of offspring overall in the mating trial within a reproductive season of *A. japonicus* . Furthermore, Table S7 shows that the higher the number of self‐cared‐for own eggs, the greater the increase in the total number of offspring of that individual. In the mating system of *A. japonicus* , males do not accept eggs being laid by females without prior copulation. Therefore, these results suggest that the number of offspring produced and male's cuckoldry rates increased with the frequency of copulation.

## 4 Discussion

Our results revealed that most males and females copulated with multiple mates, suggesting that their reproductive behavior is highly promiscuous (Figure 1). The average rate of their paternity in our experiment was 65.1%, and the observed lowest and highest rates were 10% (Male No. 1, egg mass ID: EM14) and 100% (Male No. 12, egg mass ID: EM4), respectively (Figure 2). Their paternity rates were markedly lower than originally expected (Smith 1997). In addition, the number of eggs cared for by males ranged from 18 to 111 (Figure 2).

We also estimated the cuckoldry rate of each male. The males who cared for a large number of eggs left a significantly larger number of their own offspring (Figure 3; Tables S5 and S7). This result supports the previously suggested hypothesis that males caring for more eggs, regardless of whether the eggs were fertilized by themselves or by other males, increase the number of their own fertilized eggs by enhancing mating opportunities (see Nazareth and Machado 2010; Ohba et al. 2016). In addition, most males both cuckolded other males and were cuckolded themselves (Figure 3). This phenomenon is likely a typical example of “cuckoldry,” where a male unknowingly invests resources, such as time, energy, or protection (i.e., paternal care in giant water bugs), in offspring that do not share his genetic lineage due to his mate's copulation with other males. The hypothesis proposed by Requena and Alonzo (2017) predicts that when the cost of paternal care is high and cuckoldry occurs frequently, making it difficult for males to maintain high paternity, a system that increases male mating opportunities is more likely to evolve than one that enhances male paternity. In this study, we found that in *A. japonicus* , where paternal care is costly and the promiscuous mating system leads to frequent cuckoldry, the paternal care system is still maintained, despite the low paternity rate. A similar case of paternal care evolving under conditions of cuckoldry, where paternity has been examined, is known in the black coucal *Centropus grillii* , a polyandrous bird species, in which males provide paternal care. Previous studies of *C. grillii* have reported that paternity in this species is not particularly high, with an estimated rate of about 83% (Safari and Goymann 2018; Safari et al. 2019). In contrast, the paternity rate in *A. japonicus* is approximately 65%, considerably lower than that of *C. grillii* , yet the paternal care system has still evolved and been maintained. Thus, the hypothesis proposed by Requena and Alonzo (2017) was supported by the results of our free‐choice mating trial with *A. japonicus* . In the future, conducting additional paternity analyses on species that exhibit paternal care despite a promiscuous mating system may reveal more cases supporting the hypothesis of Requena and Alonzo (2017).

Furthermore, the cuckoldry rate of Male No. 12 was particularly high; however, this male did not care for eggs fertilized by other males (Figure 3). Although Male No. 12 conducted only one paternal care cycle during the reproductive experiment, his estimated number of offspring overall in the mating trial was the third highest (Figure 3). It may be challenging to completely avoid receiving eggs fertilized by other males, and our result might be an overvaluation. This is because, in our mating trial, we did not estimate paternity for all individuals of each egg mass but instead randomly selected 10 individuals for paternity estimation. Furthermore, it is considered that if sperm competition occurs within the female's spermatheca, the male cannot fully control the fertilization process to ensure that only his sperm is used. However, it is reasonable to assume that the No. 12 male appeared to have a much lower rate of receiving such eggs compared to the other males in this experiment. Therefore, it is suggested that the number of offspring of each male (i.e., fitness) changes depending on the cuckoldry rate. In studies on the evolution of paternal care systems, genetic paternity analysis has primarily focused on fish and birds (Griffin et al. 2013). For example, in fish, the paternity rate in the pumpkinseed sunfish *Lepomis gibbosus* ranges from 43% to 100% (Rios‐Cardenas and Webster 2005), and in the bluegill sunfish *Lepomis macrochirus* , nesting males fertilize an average of 79% of the offspring in their nests (range: 26%–100%; Neff 2003). In birds, extra‐pair copulations are observed across nearly all taxa, and genetic paternity analyses have revealed that the proportion of offspring sired by extra‐pair males varies from 0% to over 65%, with an average of 15%–25% (Westneat 1995). In both fish and birds, some males achieve exceptionally high paternity, while others have low paternity, demonstrating that paternity certainty varies within species. In fish and birds, factors such as the presence of sneaker males and cooperative care by multiple males contribute to the reduced paternity of caring males (e.g., Briskie et al. 1998; Neff 2003). However, these reproductive systems differ significantly from the promiscuous mating system observed in *A. japonicus* . Although the exact reason for the exceptionally high cuckoldry rate observed in male No. 12 remains unclear, this study provides significant insights by elucidating paternity in an insect species that exhibits paternal care. Additionally, there were notable differences in the paternity and cuckoldry rates between individuals, with males who cuckolded and did not perform paternal care successfully producing offspring (Figures 2 and 3). If some males can successfully produce their offspring without actively providing paternal care, then two reproductive strategies may exist among male *A. japonicus* : one performing paternal care and one without it. In this study, we detected males successfully producing offspring by mating and depositing their sperm in females without performing paternal care. It is possible that these males exhibit a sneaker‐like strategy, similar to that observed in salmon and other fish species (Weir et al. 2016). However, whether this behavior is voluntary remains unclear, as alternative explanations, such as females refusing to lay eggs after mating, cannot be ruled out. This issue should be addressed in future research. In most species that exhibit paternal care, males do not abandon their offspring or cease providing care, even when cuckolded by other males. The lack of a clear relationship between paternity certainty and the cost of paternal care has made it challenging to understand the evolution of paternal care (Griffin et al. 2013). Based on previous studies, primarily on fish and birds, Griffin et al. (2013) suggested that males reduce paternal investment only when both the cost of paternal care is relatively high and the risk of cuckoldry is significant. However, such conditions are not met in many species, leading to the evolution of males that continue providing paternal care despite being cuckolded. For insects, other arthropods, and amphibians, where paternity analyses remain limited, accumulating individual case studies like this study will be essential for further understanding the evolutionary dynamics of paternal care.

Reportedly, the female reproductive strategy has also affected the evolution of the male's polymorphic reproductive strategies. Paternal care is essential for the hatching of eggs in giant water bugs, and eggs cannot hatch if dropped from the male's back (Ohba 2019). Males spontaneously drop their egg masses if the number of eggs in the mass is insufficient (Kight and Kruse 1992). This can be attributed to the fact that the male's cost for paternal care is too high in such cases, compared to the benefit of caring for a small number of eggs (Kight and Kruse 1992). However, individual females do not produce enough eggs in their ovaries to form a sufficiently large enough egg mass for an individual male to be willing to carry it. It is known that one giant egg mass must be formed from the eggs of multiple females (Ichikawa 1989; Ohba et al. 2016). Our parentage test also revealed that multiple females cooperated to form an egg mass (Figure 4). It was also suggested that females prefer males that care for the eggs as mating partners and add eggs to the egg masses carried by such males after copulation (Ohba et al. 2016, 2018). This behavior is thought to address the issue that a single female alone cannot lay a sufficient number of eggs for the male to engage in paternal care, as multiple females lay eggs on the back of a male that has already had eggs laid on him. We assessed this behavior as being the female's adaptive evolutionary strategy. The finding in this study that multiple females contribute to the formation of a single egg mass further supports this hypothesis. This may also select for males caring for conspecific eggs since they benefit via sexual selection.

Furthermore, females laid their eggs on the backs of numerous males (Figure 4). This behavior is considered to avoid the risk of no eggs hatching if the male dies or the quality of a particular male's paternal care is low. For males, this behavior increases the degree of promiscuity and decreases the certainty of paternity. The fact that females lay their eggs on the backs of numerous males can also preclude the risk of a termination of males' genetic line, should they themselves die during paternal care (i.e., the male's offspring are cared for by other males even if they themselves died during paternal care). The increased frequency of cuckoldry ensures that multiple males are brooding their offspring. In our mating trial experiments, the cuckoldry relationships observed between the males were highly complex (Figures 2 and 3). This is due to the promiscuous mating system of the giant water bug.

The paternal care mechanism of giant water bugs is maintained by a complex set of interactions between males and females, which has eventually led to the evolution of a highly unique combination of male reproductive strategies based on paternity and cuckoldry. In fish, paternal care has evolved in some taxa despite the absence of reversed sexual roles in courtship behavior. In these cases, male territoriality and low costs of care per brood are common traits (Reynolds et al. 2002). Additionally, in such species, paternal care may have evolved because females assess a male's caregiving ability when selecting mates (Reynolds et al. 2002). Similarly, in *A. japonicus* , males perform courtship displays, and female mate choice (i.e., selecting males that have already initiated paternal care; Ohba et al. 2016) may have driven the evolution of their unique form of paternal care, in which males carry and protect egg masses on their backs. However, in species of the subfamily Belostomatinae, the cost of paternal care is considered high (Kight et al. 1995). Despite this, paternal care has evolved, likely because the benefits of increased mating opportunities outweigh the costs, even when cuckoldry is frequent and paternity is low (see Griffin et al. 2013). Furthermore, there is limited evidence that paternal care in fish evolved primarily as a strategy to directly increase offspring survival (Goldberg et al. 2020). Instead, it is suggested that females prefer males already engaged in paternal care and the female mate choice has played a significant role in the evolution of paternal care in fish (Goldberg et al. 2020). Our study reveals that in *A. japonicus* , the paternal care system has evolved and been maintained despite low paternity rates, suggesting that female mate choice has played a significant role in the evolution of paternal care in insects as well. However, in Belostomatidae, paternal care is thought to have initially evolved in Lethocerinae species, where males protect egg masses attached to branches or other objects above the water surface (Smith 1997). To further investigate the evolutionary origins of paternal care in Belostomatidae, future studies should also examine paternity in Lethocerinae. The results produced from this study are fundamental in understanding the evolution of paternal care behavior and challenge the assumptions of past common theories that a high degree of confidence in a male's paternity is required for the evolution of paternal care (Smith 1979). Additionally, these findings support the female mate choice hypothesis (Crowl and Alexander 1989; Kight et al. 1995).

## Author Contributions

**Tomoya Suzuki:** conceptualization (equal), data curation (lead), formal analysis (equal), funding acquisition (equal), investigation (lead), project administration (equal), resources (lead), supervision (equal), visualization (lead), writing – original draft (lead), writing – review and editing (equal). **Shin‐ya Ohba:** conceptualization (equal), formal analysis (equal), funding acquisition (equal), project administration (supporting), visualization (supporting), writing – original draft (supporting), writing – review and editing (equal). **Koji Tojo:** conceptualization (equal), data curation (supporting), formal analysis (supporting), funding acquisition (equal), investigation (supporting), project administration (equal), supervision (equal), visualization (supporting), writing – original draft (supporting), writing – review and editing (equal).

## Conflicts of Interest

The authors declare no conflicts of interest.

## Supporting information


**Table S1.** Results of genotyping for *A. japonicus* using 17 microsatellite marker sets.

**Table S2.** Polymorphic information on each microsatellite locus used for the parentage test of *A. japonicus.*

**Table S3.** Results of the parentage test of *A. japonicus* in the present study.

**Table S4.** Ranking of models that explain the number of the male’s fertilized offsprings cared on the male’s dorsum (e.g., direct cared offsprings).

**Table S5.** Ranking of models that explain the number of offspring overall in the mating trial.

**Table S6.** Results from GLM on the number of the male’s fertilized offsprings cared on the male’s dorsum (e.g., direct cared offsprings).

**Table S7.** Ranking of models that explain the estimated number of offspring overall in the mating trial.

## Data Availability

All data needed to evaluate the conclusions of this study are presented in the paper and the [Link], [Link], [Link], [Link], [Link], [Link], [Link].

## References

[ece371316-bib-0001] Ah‐King, M. , C. Kvarnemo , and B. S. Tullberg . 2005. “The Influence of Territoriality and Mating System on the Evolution of Male Care: A Phylogenetic Study on Fish.” Journal of Evolutionary Biology 18: 371–382. 10.1111/j.1420-9101.2004.00823.x. 15715843

[ece371316-bib-0002] Alonzo, S. H. 2012. “Sexual Selection Favours Male Parental Care, When Females Can Choose.” Proceedings of the Royal Society B: Biological Sciences 279: 1784–1790. 10.1098/rspb.2011.2237. PMC3297463 22171082

[ece371316-bib-0003] Barton, K. , and M. K. Barton . 2013. “Package ‘MuMIn’, Version 1.18.” https://cran.r‐project.org/web/packages/MuMIn/index.html.

[ece371316-bib-0004] Briskie, J. V. , R. Montgomerie , T. Põldmaa , and P. T. Boag . 1998. “Paternity and Paternal Care in the Polygynandrous Smith's Longspur.” Behavioral Ecology and Sociobiology 43, no. 3: 181–190. 10.1007/s002650050479.

[ece371316-bib-0005] Burnham, K. , and D. Anderson . 2002. Model Selection and Multimodel Inference: A Practical Information‐Theoretic Approach. Springer.

[ece371316-bib-0006] Crowl, T. A. , and J. E. Alexander . 1989. “Parental Care and Foraging Ability in Male Water Bugs (*Belostoma flumineum*).” Canadian Journal of Zoology 67, no. 2: 513–515. 10.1139/z89-074.

[ece371316-bib-0007] Dawkins, R. 1976. The Selfish Gene. Oxford University Press.

[ece371316-bib-0008] Goldberg, R. L. , P. A. Downing , A. S. Griffin , and J. P. Green . 2020. “The Costs and Benefits of Paternal Care in Fish: A Meta‐Analysis.” Proceedings of the Royal Society B: Biological Sciences 287: 20201759. 10.1098/rspb.2020.1759. PMC7542810 32933439

[ece371316-bib-0009] Griffin, A. S. , S. H. Alonzo , and C. K. Cornwallis . 2013. “Why Do Cuckolded Males Provide Paternal Care?” PLoS Biology 11: e1001520. 10.1371/journal.pbio.1001520. 23555193 PMC3608547

[ece371316-bib-0010] Hayne, D. W. 1949. “Two Methods for Estimating Population From Trapping Records.” Journal of Mammalogy 30, no. 4: 399–411. 10.2307/1375218. 15394391

[ece371316-bib-0011] Hirshfield, M. F. , and D. W. Tinkle . 1975. “Natural Selection and the Evolution of Reproductive Effort.” Proceedings of the National Academy of Sciences of the United States of America 72: 2227–2231. 10.1073/pnas.72.6.2227. 1056027 PMC432730

[ece371316-bib-0012] Ichikawa, N. 1989. “Breeding Strategy of the Male Brooding Water Bug, Diplonychus Major Esaki (Heteroptera: Belostomatidae): Is Male Back Space Limiting?” Journal of Ethology 7: 133–140. 10.1007/BF02350035.

[ece371316-bib-0013] Inada, K. , O. Kitade , and H. Morino . 2011. “Paternity Analysis in an Egg‐Carrying Aquatic Insect *Appasus major* (Hemiptera: Belostomatidae) Using Microsatellite DNA Markers.” Entomological Science 14: 43–48. 10.1111/j.1479-8298.2010.00420.x.

[ece371316-bib-0014] Jones, A. G. , and J. C. Avise . 2001. “Mating Systems and Sexual Selection in Male‐Pregnant Pipefishes and Seahorses: Insights From Microsatellite‐Based Studies of Maternity.” Journal of Heredity 92: 150–158. 10.1093/jhered/92.2.150. 11396573

[ece371316-bib-0015] Kalinowski, S. T. , M. L. Taper , and T. C. Marshall . 2007. “Revising How the Computer Program CERVUS Accommodates Genotyping Error Increases Success in Paternity Assignment.” Molecular Ecology 16: 1099–1106. 10.1111/j.1365-294X.2007.03089.x. 17305863

[ece371316-bib-0016] Kamel, S. J. , and R. K. Grosberg . 2012. “Exclusive Male Care Despite Extreme Female Promiscuity and Low Paternity in a Marine Snail.” Ecology Letters 15: 1167–1173. 10.1111/j.1461-0248.2012.01841.x. 22834645

[ece371316-bib-0017] Kight, S. L. , and K. C. Kruse . 1992. “Factors Affecting the Allocation of Paternal Care in Waterbugs (*Belostoma flumineum* say).” Behavioral Ecology and Sociobiology 30, no. 6: 409–414. 10.1007/BF00176176.

[ece371316-bib-0018] Kight, S. L. , J. Sprague , K. C. Kruse , and L. Johnson . 1995. “Are Egg‐Bearing Male Water Bugs, *Belostoma flumineum* Say (Hemiptera: Belostomatidae), Impaired Swimmers?” Journal of the Kansas Entomological Society 68: 468–470.

[ece371316-bib-0019] Kokko, H. , and M. Jennions . 2003. “It Takes Two to Tango.” Trends in Ecology & Evolution 18: 103–104. 10.1016/S0169-5347(03)00009-0.

[ece371316-bib-0020] Kokko, H. , and M. D. Jennions . 2008. “Parental Investment, Sexual Selection and Sex Ratios.” Journal of Evolutionary Biology 21: 919–948. 10.1111/j.1420-9101.2008.01540.x. 18462318

[ece371316-bib-0021] Kölliker, M. , P. T. Smiseth , and N. J. Royle . 2012. “Evolution of Parental Care.” In The Princeton Guide to Evolution, edited by J. B. Losos , D. A. Baum , D. J. Futuyma , et al., 663–669. Princeton University Press.

[ece371316-bib-0022] Lehtinen, R. M. , and R. A. Nussbaum . 2003. “Parental Care: A Phylogenetic Perspective.” In Reproductive Biology and Phylogeny of Anura, edited by B. G. M. Jamieson , 343–386. Science Publishers Inc.

[ece371316-bib-0023] Nazareth, T. M. , and G. Machado . 2010. “Mating System and Exclusive Postzygotic Paternal Care in a Neotropical Harvestman (Arachnida: Opiliones).” Animal Behaviour 79: 547–554. 10.1016/j.anbehav.2009.11.026.

[ece371316-bib-0024] Neff, B. D. 2003. “Paternity and Condition Affect Cannibalistic Behavior in Nest‐Tending Bluegill Sunfish.” Behavioral Ecology and Sociobiology 54: 377–384. 10.1007/s00265-003-0645-9.

[ece371316-bib-0025] Ohba, S. 2019. “Ecology of Giant Water Bugs (Hemiptera: Heteroptera: Belostomatidae).” Entomological Science 22: 6–20. 10.1111/ens.12334.

[ece371316-bib-0026] Ohba, S. , S. Matsuo , T. T. T. Huynh , and S. Kudo . 2018. “Female Mate Preference for Egg‐Caring Males in the Giant Water Bug *Diplonychus rusticus* (Heteroptera Belostomatidae).” Ethology Ecology & Evolution 30, no. 5: 477–484. 10.1080/03949370.2018.1438517.

[ece371316-bib-0027] Ohba, S. , N. Okuda , and S. Kudo . 2016. “Sexual Selection of Male Parental Care in Giant Water Bugs.” Royal Society Open Science 3: 150720. 10.1098/rsos.150720. 27293778 PMC4892440

[ece371316-bib-0028] Requena, G. S. , and S. H. Alonzo . 2017. “Sperm Competition Games When Males Invest in Paternal Care.” Proceedings of the Royal Society B: Biological Sciences 284: 20171266. 10.1098/rspb.2017.1266. PMC5563816 28814658

[ece371316-bib-0029] Reynolds, J. D. , N. B. Goodwin , and R. P. Freckleton . 2002. “Evolutionary Transitions in Parental Care and Live Bearing in Vertebrates.” Philosophical Transactions of the Royal Society of London. Series B: Biological Sciences 357, no. 1419: 269–281. 10.1098/rstb.2001.0930. 11958696 PMC1692951

[ece371316-bib-0030] Ridley, M. W. 1978. “Paternal Care.” Animal Behaviour 26: 904–932. 10.1016/0003-3472(78)90156-2.

[ece371316-bib-0031] Rios‐Cardenas, O. , and M. S. Webster . 2005. “Paternity and Paternal Effort in the Pumpkinseed Sunfish.” Behavioral Ecology 16: 914–921. 10.1093/beheco/ari076.

[ece371316-bib-0032] Rueger, T. , H. B. Harrison , N. M. Gardiner , M. L. Berumen , and G. P. Jones . 2019. “Extra‐Pair Mating in a Socially Monogamous and Paternal Mouth‐Brooding Cardinalfish.” Molecular Ecology 28: 2625–2635. 10.1111/mec.15103. 30985980

[ece371316-bib-0033] Safari, I. , and W. Goymann . 2018. “Certainty of Paternity in Two Coucal Species With Divergent Sex Roles: The Devil Takes the Hindmost.” BMC Evolutionary Biology 18: 110. 10.1186/s12862-018-1225-y. 30005606 PMC6043945

[ece371316-bib-0035] Safari, I. , W. Goymann , and H. Kokko . 2019. “Male‐Only Care and Cuckoldry in Black Coucals: Does Parenting Hamper Sex Life?” Proceedings of the Royal Society B: Biological Sciences 286: 20182789. 10.1098/rspb.2018.2789. PMC6501686 30966989

[ece371316-bib-0036] Skinner, B. F. 1981. “Selection by Consequences.” Science 213: 501–504. 10.1126/science.7244649. 7244649

[ece371316-bib-0037] Smith, R. L. 1979. “Repeated Copulation and Sperm Precedence: Paternity Assurance for a Male Brooding Water Bug.” Science 205: 1029–1031. 10.1126/science.205.4410.1029. 17795564

[ece371316-bib-0038] Smith, R. L. 1997. “Evolution of Paternal Care in the Giant Water Bugs (Heteroptera: Belostomatidae).” In The Evolution of Social Behavior in Insects and Arachnids, edited by J. C. Choe and B. J. Crespi , 116–149. Cambridge University Press.

[ece371316-bib-0039] Suzuki, T. , A. S. Hirao , M. Takenaka , K. Yano , and K. Tojo . 2021. “Development of Microsatellite Markers for a Giant Water Bug, *Appasus japonicus* , Distributed in East Asia.” Genes & Genetic Systems 95: 323–329. 10.1266/ggs.20-00033. 33487614

[ece371316-bib-0040] Tallamy, D. W. 2001. “Evolution of Exclusive Paternal Care in Arthopods.” Annual Review of Entomology 46: 139–165. 10.1146/annurev.ento.46.1.139. 11112166

[ece371316-bib-0041] Valencia‐Aguilar, A. , K. R. Zamudio , C. F. B. Haddad , S. M. Bogdanowicz , and C. P. A. Prado . 2020. “Show Me You Care: Female Mate Choice Based on Egg Attendance Rather Than Male or Territorial Traits.” Behavioral Ecology 31: 1054–1064. 10.1093/beheco/araa051.

[ece371316-bib-0042] Van Oosterhout, C. , W. F. Hutchinson , D. P. M. Wills , and P. Shipley . 2004. “MICRO‐CHECKER: Software for Identifying and Correcting Genotyping Errors in Microsatellite Data.” Molecular Ecology Notes 4: 535–538. 10.1111/j.1471-8286.2004.00684.x.

[ece371316-bib-0043] Venables, W. , and B. Ripley . 2002. “Spatial Statistics.” In Modern Applied Statistics With S, edited by B. D. Ripley , 4th ed., 419–434. Springer.

[ece371316-bib-0044] Weir, L. K. , H. K. Kindsvater , K. A. Young , and J. D. Reynolds . 2016. “Sneaker Males Affect Fighter Male Body Size and Sexual Size Dimorphism in Salmon.” American Naturalist 188: 264–271. 10.1086/687253. 27420790

[ece371316-bib-0045] Westneat, D. F. 1995. “Paternity and Paternal Behaviour in the Red‐Winged Blackbird, *Agelaius phoeniceus* .” Animal Behaviour 49: 21–35. 10.1016/0003-3472(95)80150-2.

